# Rates and risk factors of oocyte immaturity: toward personalized selection for rescue in vitro maturation

**DOI:** 10.1007/s10815-025-03722-z

**Published:** 2025-11-14

**Authors:** Marilena Taggi, Roberta Maggiulli, Federica Innocenti, Valentina Casciani, Greta Chiara Cermisoni, Daria Maria Soscia, Pasquale Petrone, Alessandro Ruffa, Laura Albricci, Giulia Fiorentino, Maurizio Zuccotti, Alberto Vaiarelli, Antonio Capalbo, Giovanni Coticchio, Laura Rienzi, Danilo Cimadomo

**Affiliations:** 1 IVIRMA Global Research Alliance, Genera, Clinica Valle Giulia, Rome, Italy; 2 Department of Biomedicine and Prevention, University Tor Vergata, Rome, Italy; 3 IVIRMA Global Research Alliance, IVI, Roma, Rome, Italy; 4 Department of Biology and Biotechnology ‘Lazzaro Spallanzani’, Laboratory of Biology and Biotechnology of Reproduction, University of Pavia, Pavia, Italy; 5 Juno Genetics, Rome, Italy; 6 Unit of Molecular Genetics, Center for Advanced Studies and Technology (CAST), “G. d’Annunzio” University of Chieti-Pescara, Chieti, Italy; 7 IVIRMA Global Research Alliance, IVIRMA Italia, Rome, Italy; 8 Department of Biomolecular Sciences, University of Urbino “Carlo Bo”, Urbino, Italy

**Keywords:** Immature oocytes, Rescue IVM, Maturation rate, Ovarian stimulation, Standard operating procedures

## Abstract

**Purpose:**

To identify treatment-related factors influencing low oocyte maturation rates in standard ovarian stimulation (OS) cycles and patients’ candidates for clinical studies on rescue-IVM.

**Methods:**

Retrospective study including retrievals with ≥ 1 cumulus-oocyte-complex (COC; years: 2008–2022). The weighted-mean immaturityrate (19%) was defined in the whole dataset (*N* = 16,155). Variables associated with immaturity rates and warning limit (defined as weighted average + 2SD) were appraised among first retrievals with ≥ 5 COCs (*N* = 7962). Of the patients undergoing a first oocyte pick-up, 667 completed three retrieval cycles, enabling evaluation of the true prevalence of patients exceeding the immaturity rate warning threshold over multiple cycles.

**Results:**

Factors influencing immaturity rate included OS duration, trigger type (GnRH-agonist versus urinary-hCG), ovulation trigger to oocytes’ denudation interval and ratio COC to follicle > 14 mm at ovulation trigger. In first retrievals with ≥ 5 COCs, the immaturity rate warning limit was 51%, occurring in 3.6% of initial retrievals, 3.8% of second retrievals, and 2.1% of third retrievals. In three consecutive retrievals, the conservative prevalence of patients exceeding this threshold once, twice, and three times was 4.4%, 0.3%, and 0.03%, respectively. Assuming all patients would have conducted three retrievals, these rates were estimated as 7.8%, 1.5%, and 0.3%. In the 667 patients who conducted three retrievals, observed rates were 7.6%, 0.9%, and 0.4%, confirming the reliability of the estimates.

**Conclusions:**

Ovarian stimulation and laboratory factors impact oocyte maturation rate. An oocyte immaturity rate exceeding 51%, in patients retrieving ≥ 5 oocytes, may represent a strong inclusion criterion for future clinical studies on rescue-IVM.

**Supplementary information:**

The online version contains supplementary material available at 10.1007/s10815-025-03722-z.

## Introduction

The oocyte plays a critical role in determining the developmental competence of the embryo. In addition to providing half of the chromosomal complement, it also provides all the cytoplasmic material, which contains mitochondria, mRNA, proteins, and essential nutrients—all of which are crucial for the early stages of embryonic development [1]. Oocyte maturation involves the coordination of mutually integrated cytoplasmic and nuclear events. In the nucleus, this process includes germinal vesicle breakdown (GVBD), the resumption of meiosis, and the completion of the first meiotic division. Simultaneously, cytoplasmic maturation encompasses the redistribution of organelles, the establishment of oocyte polarity, and the accumulation of mRNA, proteins, and nutrients essential for achieving the developmental competence required for proper embryonic development [2, 3]. While defining cytoplasmic maturation is a challenge, nuclear maturation is denoted by the extrusion of the first polar body (PB1).

In ART, a well-designed ovarian stimulation (OS) protocol is essential to promote the coordinated growth of multiple follicles, increasing the number of collected oocytes and therefore embryos available for transfer and ultimately maximizing pregnancy potential. However, approximately 10–20% of oocytes retrieved after OS remain immature, arrested at either the metaphase I (MI) or germinal vesicle (GV) stages [4, 5], failing to progress to the metaphase II (MII) stage. The exact mechanisms leading to failed oocyte maturation in IVF cycles are not fully understood. In recent years, scientists have explored the specific conditions of oocyte maturation arrest (OMA) and identified several relevant genes. Studies involving whole-exome sequencing (WES) in infertile women have revealed mutations in genes that play crucial roles in meiosis, spindle formation, and cytoplasmic maturation. These genetic insights have shed light on the molecular basis of maturation failures and opened new avenues for understanding infertility derived from oocyte defects [6, 7]. Oocyte maturation failure may also be caused by non-genetic factors; specifically, perturbations in follicle growth during ovarian stimulation may uncouple the mechanisms coordinating follicle ovulation and oocyte maturation, leading to oocyte inability to resume and complete the GV-MII transition [8].

Oocyte in vitro maturation (IVM), also referred to as standard IVM, is defined as the laboratory process of maturing immature cumulus–oocyte complexes (COCs) obtained from antral follicles [9]. This technology entails the collection from mid-antral follicles and subsequent culture of immature oocytes at the GV stage, still enclosed in their cumulus cells (CCs), to achieve in vitro meiotic maturity at the MII stage. This approach is particularly beneficial for patients with polycystic ovary syndrome (PCOS) or premature ovarian failure (POF), as it allows oocytes to mature without prior sustained hormonal stimulation, thereby reducing the associated risks and/or improving treatment acceptance by these patient populations [10–12].

In conventional ART cycles, immature oocytes are generally discarded due to their presumed unsuitability for treatment purposes. Nonetheless, in patients with diminished ovarian reserve (DOR) or poor ovarian response (POR) to hormonal stimulation, protocols aimed at promoting meiotic and developmental competence to these immature oocytes might be advantageous. Rescue-IVM, which involves the culture of denuded immature oocytes, at the GV or MI stage, collected from conventional ovarian stimulation cycles, has been recommended by the ASRM as potentially beneficial for patients with a poor prognosis [13]. However, the clinical use of immature oocytes remains a subject of debate. Today, even the Istanbul consensus advocates for considering rescue-IVM as a viable option for poor-prognosis patients and for patients with an unsynchronized follicle cohort [14], potentially opening opportunities for the future.

Considered the lack of precise guidelines in this field, our study aims at determining a statistical cut-off of the rate of oocyte immaturity: this would provide an estimate of the patient population affected by this phenomenon and an objective criterion for targeted clinical approaches, such as rescue-IVM. The knowledge gained from this approach may provide crucial information on the causes of maturation defects and enable the development of new, personalised clinics.

## Materials and methods

### Study design

This retrospective study includes 16,155 IVF cycles using own gametes, with each cycle involving the recovery of at least one cumulus–oocyte complex (COC). The study was conducted at a private IVF center in Rome between 2008 and 2022. Ethical committee approval was obtained for the retrospective analysis of pseudonymized data, aimed at identifying patient, cycle, or embryo characteristics associated with IVF efficacy and efficiency.

From the 16,155 oocyte pick-ups (OPUs) following OS, we calculated the weighted average oocyte immaturity rate, calculated as the sum of each value (i.e., immaturity rate) multiplied by its weight (i.e., COCs retrieved), divided by the sum of the weights (i.e., COCs retrieved). To analyze potential variables affecting oocyte immaturity rates (detailed in Table 1), we focused on first OPUs from couples collecting at least five COCs (*N* = 7962), and ran regression analyses. These factors include maternal characteristics (age, karyotype, body mass index, infertility [kind/main cause/duration], hormonal levels) and cycle parameters such as OS features (protocol/length/total dose/gonadotrophin used, kind and dose of ovulation trigger), time intervals (ovulation trigger to oocytes’ denudation/ovulation trigger to OPU/OPU to oocytes’ denudation), number of follicles > 14 mm the day of ovulation trigger, and ratio COC to follicle > 14 mm at ovulation trigger. The main cause of female infertility was classified as idiopathic in women younger than 35 years with AMH levels above 1.2 ng/ml and no identifiable underlying reason. Infertility because of endocrine-ovulatory dysfunctions, tubal factor or endometriosis was defined independently of woman age. Idiopathic infertility in women ≥ 35 years old was defined as advanced maternal age (AMA) independently of AMH, whereas DOR characterizes women younger than 35 years with AMH levels below 1.2 ng/ml.

**Table 1:** Description of the population of patients undergoing their first oocyte pick up (OPU) and collecting ≥ 5 cumulus oocyte complexes (COCs)

	First OPUs with ≥ 5 COCs ***N*** = 7962
**Maternal age** *median (Q1, Q3)*	37.0 (34, 40) years
**Maternal karyotype** **46, XX** *N (%)* **Mosaic or sex chromosome abnormality** *N (%)* **Structural abnormality** *N (%)*	*N* = 7820, 98.2% *N* = 60, 0.8% *N* = 82, 1%
**Maternal BMI** *median (Q1, Q3)*	21.5 (19.7, 23.8) kg/m^2^
**FSH** *median (Q1, Q3)*	8.9 (6.4, 12.9) mIU/ml
**AMH** *median (Q1, Q3)*	1.9 (1.1, 2.8) ng/ml
**Previous conception(s)** **No** *N (%)* **Yes** *N (%)*	*N* = 2078, 26.1% *N* = 5884, 73.9%
**Main cause of female infertility** **Idiopathic** *N (%)* **Endocrine ovulatory** *N (%)* **Tubal** *N (%)* **Endometriosis** *N (%)* **Advanced maternal age** *N (%)* **Diminished ovarian reserve** *N (%)*	*N* = 1168, 14.7% *N* = 254, 3.2% *N* = 734, 9.2% *N* = 405, 5.1% *N* = 4978, 62.5% *N* = 423, 5.3%
**Years of infertility** *median (Q1, Q3)*	3 (1, 4) years
**OS protocol** **FSH + antagonist** *N (%)* **Long** *N (%)* **Short flare-up** *N (%)*	*N* = 6319, 79.4% *N* = 1109, 13.9% *N* = 534, 6.7%
**OS duration*** median (Q1, Q3)*	11 (10, 12) days
**OS total gonadotrophins dose** ** < 1500 IU** *N (%)* **1500–2500 IU** *N (%)* **2500–3000 IU** *N (%)* **3000–4000 IU** *N (%)* **4000–5000 IU** *N (%)* ** > 5000 IU** *N (%)*	*N* = 1097, 13.8% *N* = 2339, 29.4% *N* = 1585, 19.9% *N* = 1920, 24.1% *N* = 972, 12.2% *N* = 49, 0.6%
**Trigger type** **Urinary-hCG** *N (%)* **GnRH-agonist** *N (%)*	*N* = 4418, 55.5% *N* = 3544, 44.5%
**Ovulation trigger to oocytes’ denudation interval** *median (Q1, Q3)*	39.1 (38.2, 40.1) hours
**Ovulation trigger to OPU interval*** median (Q1, Q3)*	35.4 (35.2, 35.6) hours
**OPU to oocytes’ denudation interval*** median (Q1, Q3)*	3.6 (2.8, 4.6) hours
**Follicles > 14 mm at ovulation trigger*** median (Q1, Q3)*	9 (6, 14)
**COCs*** median (Q1, Q3)*	10 (7, 14)
**Ratio COCs to follicles > 14 mm at ovulation trigger*** median (Q1, Q3)*	1.13 (0.9, 1.4)

*BMI* body mass index, *OS* ovarian stimulation

Finally, we used the dataset of first retrievals with at least 5 COCs to outline a statistically robust warning limit. Warning limits are commonly used to monitor key performance indicators (KPIs) as part of a clinic’s quality management system. These limits are calculated as weighted average ± 2 SD [15–17]. This approach allows for the early detection of unusual fluctuations, preventing them from reaching critical levels and enabling the implementation of corrective actions. It thus provides a sensitive and stable method for performance monitoring, ensuring process control and consistency. In this study, we postulate that the upper warning limit of immaturity rates after OS can be leveraged to candidate patients for clinical investigations of rescue-IVM.

Next, we calculated the conservative, estimated and true prevalences of patients exceeding the warning limit for oocyte immaturity rates across multiple retrievals. The conservative prevalence reflects the actual observed data, derived from all patients who underwent at least one OPU with ≥ 5 COCs. It measures how many patients surpassed the warning limit once, twice, or three times. The estimated prevalence modelled a scenario in which all patients underwent three consecutive cycles, by applying the observed oocyte immaturity rates from the second and third retrievals to patients who did not complete them. The true prevalence summarized the data from the 667 patients who indeed underwent three OPUs between 2008 and 2022, by calculating the true proportion of these patients who exceeded the warning limit once, twice, or three times.

**Fig. 1:**
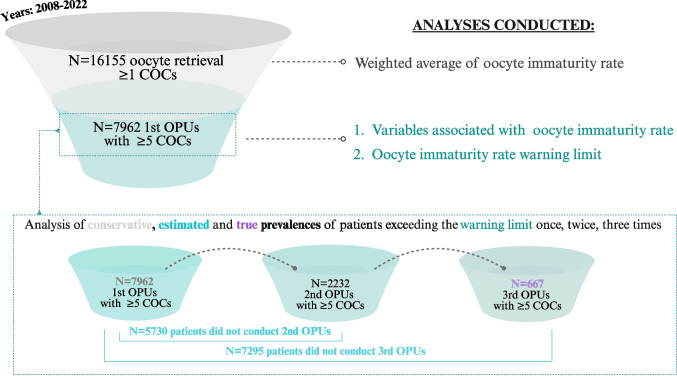
Study flowchart. The weighted-average immaturity rate and outliers for cumulus oocytes complexes (COCs) retrieved were defined across the entire dataset (*N* = 16,155). Variables influencing immaturity rates were examined among first retrievals with ≥ 5 COCs (*N* = 7962). In the same dataset, we outlined the warning limit for the immaturity rate. Lastly, the conservative, estimated, and true prevalence of immaturity rates exceeding the warning limit across multiple retrievals were calculated as detailed in the materials and methods section of the manuscript. COCs, cumulus-oocyte complexes; OPU, oocyte pick-up

The study flowchart summarizing these analyses is presented in Fig. 1.

### IVF procedures

Patients underwent OS for IVF with different protocols, including long GnRH agonist, GnRH antagonist, and microdose GnRH agonist flare-up. Different types of gonadotropins were used, including recombinant-FSH with or without recombinant-LH, urinary HMG or corifollitropin alpha. The type and dose of gonadotropin were selected based on patient characteristics (age, ovarian reserve markers, and compliance with the method of administration) and the gynaecologist’s evaluation aimed at optimizing the number of oocytes retrieved per OS. Briefly, three different dosages were used based on ovarian reserve and expected ovarian response to OS, i.e., 150 IU/day, 225 IU/day, and 300 IU/day for expected high, normal, and poor responders, respectively. LH and/or hCG activity preparations (i.e., rec-LH or HMG) were administered to patients with expected sub-optimal or poor response with rec-FSH monotherapy. After the scan and basal assessment of the ovaries, the OS started in the early follicular phase (day 2 of the menstrual cycle). Transvaginal ultrasound monitoring was conducted every 2–5 days to assess follicular growth. The mean diameter of all the follicles ≥ 10 mm was recorded in the database. In the antagonist protocol, daily subcutaneous injections of GnRH antagonist—either cetrorelix 0.25 mg or ganirelix 0.25 mg—were added when the follicular diameter exceeded 13 mm until and including the day of the trigger. Once at least three follicles reached an average diameter ≥ 18 mm, final follicular maturation was induced through either urinary-hCG 10,000 IU or GnRH-agonist with 0.3 mg of triptorelin acetate or a single subcutaneous bolus of buserelin 50 IU. The GnRH-agonist trigger was used in case of expected hyper-response and in all cases when a freeze-all strategy was planned, such as when the endometrium was inadequate or when preimplantation genetic testing (PGT) was planned. OPU was performed on average 35 h after the trigger. Before proceeding with OPU, the accuracy of the trigger administration (including the dosage, type of medication, and the precise time of ovulation trigger) was confirmed by the patient to the embryologist and noted in the database. Every follicle above 10 mm was aspirated under transvaginal ultrasound guidance. Cumulus-oocyte complexes (COCs) were placed in culture medium containing human serum albumin and incubated at 37 °C, 6%CO_2_, 5%O_2_, 95% relative humidity for at least 2 h before cumulus and corona cells removal. Denudation was performed by initially exposing the oocytes to a buffered medium containing 80 IU/ml of hyaluronidase for < 1 min and gentle pipetting with a glass Pasteur pipette to remove most of the cumulus cells. The denudation was completed in hyaluronidase-free medium by stripping the remaining cumulus and corona cells with denuding pipettes of 170-mm and 140-mm diameters. Rescue-IVM was not conducted, and immature oocytes were discarded or donated for research.

### Statistical analysis

The software SPSS version 29 was used for statistics (IBM, USA). Continuous data are shown as mean ± SD. The normal (Gaussian) distribution of the data was assessed with a Shapiro–Wilk test and either *t*-test/ANOVA or Mann–Whitney *U*/Kruskal Wallis tests were adopted to assess statistically significant differences. Fisher’s exact/chi-squared test was instead adopted for categorical variables. Binomial regression with a logit link function was used to model the immaturity rate formulated as the number of events (immature oocytes) per cohort (total collected oocytes) versus women and cycle characteristics. Non-linear associations were modelled with restricted cubic splines and model fits were compared using the Akaike information criterion. Predicted marginal means from multivariable models were plotted to depict the associations.

## Results

### Weighted-average oocyte immaturity rate

Using the original dataset (16,155 OPUs, each involving at least one retrieved oocyte), we calculated the weighted average immaturity rate. Our analysis revealed an immaturity rate of 19%, suggesting that normally ≥ 1 immature oocyte might be obtained with ≥ 5 COCs (Supplementary Fig. 1). Focusing on the specific stage of meiotic arrest, we distinguished the proportion of oocytes at the GV stage from those at the MI stage. As shown in Supplementary Fig. 1, the GV oocytes’ weighted rate was 12%, while the MI oocytes’ weighted rate was 7%.

### Variables associated with oocyte immaturity rate

**Fig. 2:**
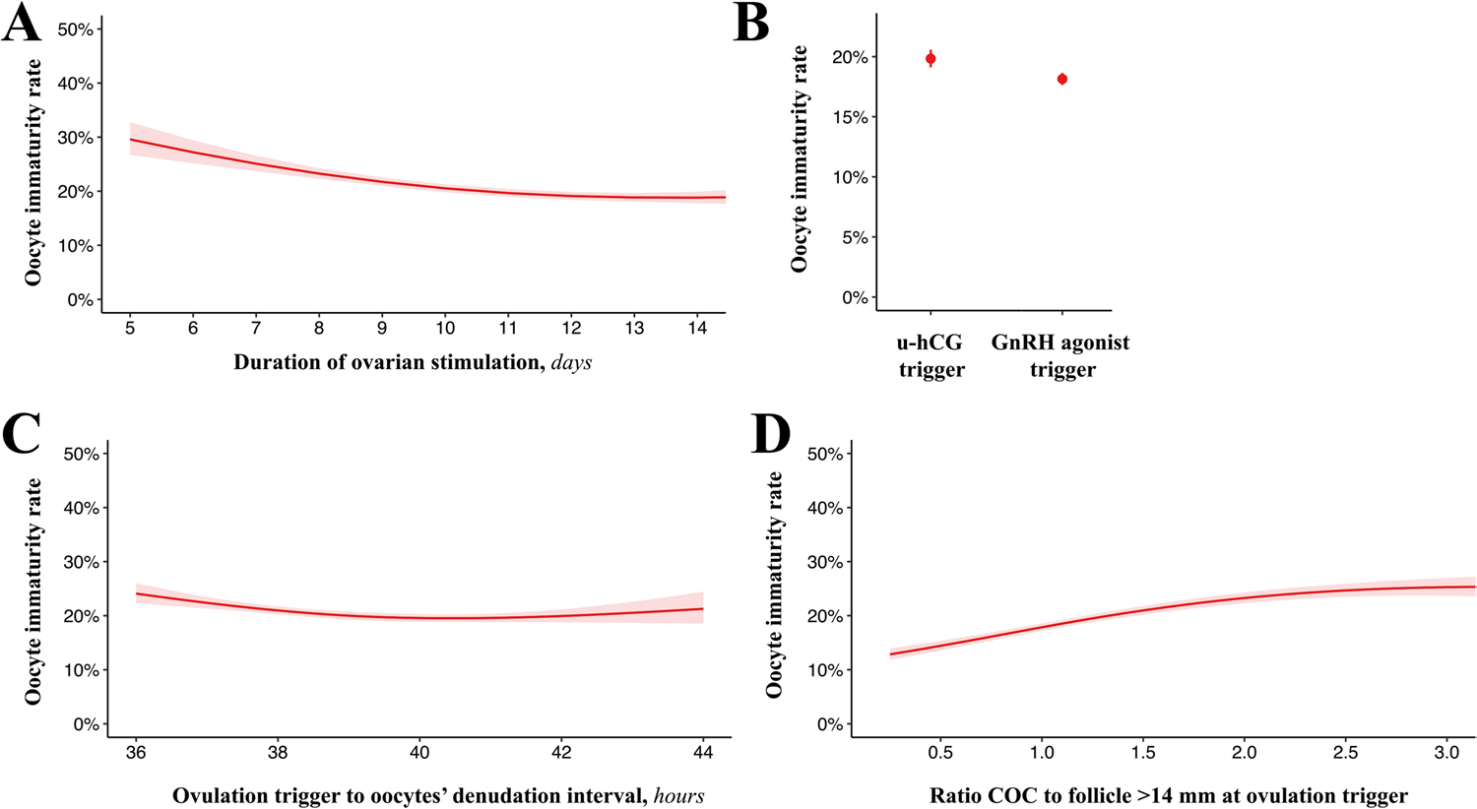
Variables associated with oocyte immaturity rates. The variables significantly associated with immaturity rates include ovarian stimulation length (**A**), the use of a GnRH agonist trigger versus urinary hCG (u-hCG) (**B**), the ovulation trigger to oocytes’ denudation interval (**C**), and the ratio of cumulus oocyte complex (COC) to follicle > 14 mm at ovulation trigger (**D**). Binomial regression with logit link function was used to model the immaturity rate formulated as number of events (immature oocytes) per cohort (total collected oocytes) versus the characteristics under investigation. Non-linear associations were modelled with restricted cubic splines and model fits were compared using the Akaike information criterion. Predicted marginal means from multivariable models were plotted to depict the associations

No patient characteristic showed significant associations with oocyte immaturity rate (Supplementary Fig. 2). Conversely, among all the cycles characteristics tested, only four showed significant associations with this outcome, namely duration of OS, trigger type, ovulation trigger to oocytes’ denudation interval, and ratio COC to follicle > 14 mm at ovulation trigger (Fig. 2A–D, Supplementary Tables 1–4, Table 2). In detail, OS shorter than 11 days showed a progressive reduction of oocyte immaturity rates from ~ 30% to ~ 20% (univariate RR 0.44, 95% CI 0.39–0.51, *p* < 0.001; multivariate RR 0.45, 95% CI 0.38–0.54, *p* < 0.001; Supplementary Table 1, Table 2, Fig. 2A) to then plateau in OS lasting 11 to 14 days (univariate RR 0.92, 95% CI 0.78–1.09, *p* = 0.344; multivariate RR 0.89, 95% CI 0.71–1.11, *p* = 0.306; Supplementary Table 1, Table 2, Fig. 2A). Regarding trigger type, use of GnRH-agonist resulted in a modest but statistically significant reduction in oocyte immaturity rates compared to urinary-hCG (univariate RR 0.91, 95% CI 0.88–0.93, *p* < 0.001; multivariate RR 0.92, 95% CI 0.88–0.95, *p* < 0.001; Supplementary Table 2, Table 2, Fig. 2B). The third relevant variable was time; specifically, ovulation trigger to oocytes’ denudation intervals between 36 and 39 h were associated with a decreasing risk of oocyte immaturity from ~ 25% to ~ 20% (univariate RR 0.77, 95% CI 0.67–0.88, *p* < 0.001; multivariate RR 0.69, 95% CI 0.60–0.80, *p* < 0.001; Supplementary Table 3, Table 2, Fig. 2C), while this rate was rather stable in the range 39–44 h (univariate RR 1.00, 95% CI 0.87–1.15, *p* = 0.987; multivariate RR 0.99, 95% CI 0.86–1.13, *p* = 0.870; Supplementary Table 3, Table 2, Fig. 2C). This effect was the contribution of both intermediate intervals, namely ovulation trigger to OPU (ranging 34 to 38 h; Supplementary Fig. 3A) and OPU to oocytes’ denudation (ranging 2–6 h; Supplementary Fig. 3B). Lastly, a ratio COC to follicle > 14 mm at ovulation trigger in the range 0.25–1.13 was associated with a markedly increasing risk of oocyte immaturity (univariate RR 2.90, 95% CI 2.54–3.32, *p* < 0.001; multivariate RR 3.11, 95% CI 2.63–3.67, *p* < 0.001; Supplementary Table 4, Table 2, Fig. 2D), which kept increasing also in the range 1.13–4 but with a less pronounced slope (univariate RR 1.68, 95% CI 1.52–1.84, *p* < 0.001; multivariate RR 1.52, 95% CI 1.34–1.71, *p* < 0.001; Supplementary Table 4, Table 2, Fig. 2D).

**Table 2:** Binomial regression with logit link function to model the immaturity rate formulated as number of events (immature oocytes) per cohort (total collected oocytes) versus all variables that were significant from the univariate analyses. Non-linear associations were modelled with restricted cubic splines and model fits were compared using Akaike information criterion

*Predictors*	*Risk ratio*	*95%CI*	*p-value*
(Intercept)	0.23	0.20–0.27	** < 0.001**
Duration of ovarian stimulation [< 11 days]	0.45	0.38–0.54	** < 0.001**
Duration of ovarian stimulation [> 11 days]	0.89	0.71–1.11	0.306
Trigger type [GnRH agonist versus urinary hCG]	0.92	0.88–0.95	** < 0.001**
Ovulation trigger to oocytes’ denudation interval [< 39 h]	0.69	0.60–0.80	** < 0.001**
Ovulation trigger to oocytes’ denudation interval [> 39 h]	0.99	0.86–1.13	0.870
Ratio COC to fol1icle > 14 mm at ovulation trigger [< 1.13]	3.11	2.63–3.67	** < 0.001**
Ratio COC to fol1icle > 14 mm at ovulation trigger [> 1.13]	1.52	1.34–1.71	** < 0.001**

The same variables analyzed for the overall oocyte immaturity rate were also evaluated separately for GV and MI oocytes’ rates. While for the MI rate, all previous associations were confirmed, for the GV rate, the ovulation trigger to oocytes’ denudation interval showed no association (Supplementary Figs. 4, and 5).

### Definition of oocyte immaturity rate warning limit

The warning limit of oocyte immaturity rates was 51% among the 7962 first retrievals with ≥ 5 COCs. This event was reported in 3.6% (*N* = 286/7962) of the first OPUs with at least 5 COCs, 3.8% (*N* = 86/2232) of the second OPUs, and 2.1% (*N* = 14/667) of the third OPUs.

The conservative prevalence of patients exceeding this warning limit in one, two, or three OPUs was 4.4% (*N* = 353/7962), 0.3% (*N* = 23/7962), and 0.03% (*N* = 2/7962), respectively. Assuming all 7962 patients would conduct three consecutive retrievals, we calculated the estimated prevalence as 7.8% (*N* = 353 + 257/7962), 1.5% (*N* = 23 + 93/7962), and 0.3% (*N* = 2 + 21/7962), respectively. Lastly, among the 667 patients who indeed underwent three consecutive retrievals, the true prevalence was 7.6%, 0.9%, and 0.4%, respectively. These last percentages align with the estimated prevalence, in turn supporting the reliability of these projections (Table 3).

**Table 3:**
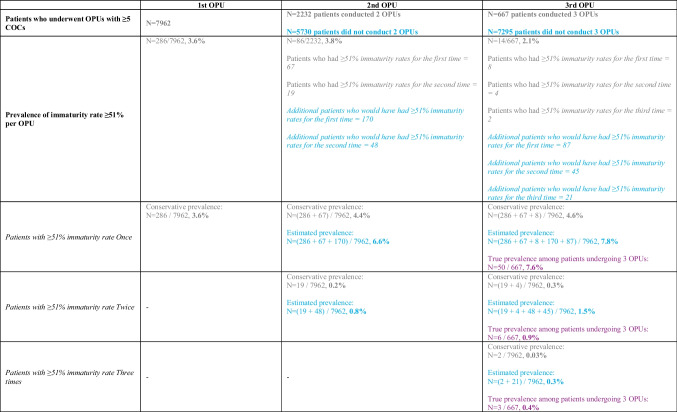
Evaluation of the conservative prevalence (in grey) estimated prevalence (in blue), and true prevalence (in purple) of immaturity rates exceeding the warning limit across multiple retrievals

*COCs* cumulus-oocyte complexes, *OPU* oocyte pick-up

## Discussion

The results of this study provide new insights into the assessment and possible causes of oocyte immaturity rates following OS, with potential implications for future clinical practice. Our data indicate an overall oocyte immaturity rate of 19%, with a higher incidence of arrest at the GV stage (12%) compared to the MI stage (7%). Several studies suggest that in vitro matured MII originating from GV oocytes exhibit lower developmental competence than those derived from MI oocytes. However, this competence remains reasonably high when MI oocytes mature in vitro within 6 h but is significantly compromised if MII oocytes originate from GV or MI oocytes that mature overnight [5, 18, 19]. Nevertheless, when blastocysts are successfully obtained, they generally exhibit aneuploidy rates comparable to sibling MII oocytes, regardless of their origin [20, 21]. These findings underscore the importance of differentiated clinical management strategies for GV and MI oocytes in rescue-IVM protocols, considering their distinct physiological characteristics to optimize maturation outcomes. Furthermore, recent evidence indicates that oocyte developmental competence varies with advancing maternal age: while MII oocytes exhibit a marked decline in quality, GV oocytes demonstrate improved potential, and MI oocytes remain relatively unchanged [22]. These findings challenge the long-standing assumption that oocyte maturity grades are functionally static across the reproductive lifespan. Although maternal age is a non-modifiable variable, its impact necessitates a re-evaluation of stimulation protocols, OPU timing, and the clinical management of immature oocytes, particularly GV-stage oocytes in older patients or those with DOR. Overall, the results of this study underscore the clinical relevance of assessing oocyte immaturity patterns, suggesting that tailored strategies based on meiotic stage and patient characteristics may optimize maturation outcomes and improve IVF success. For instance, although our analysis did not reveal a significant association between the main cause of female infertility and oocyte immaturity rate, DOR patients showed the lowest median rate. This observation is imputable to these patients having a median ratio COC to follicle > 14 mm at ovulation trigger of 1 (Q1 = 0.8, Q3 = 1.25). In other terms, DOR women obtain less mature follicles at the end of OS and most of the COCs are retrieved from these follicles, thus being subject to a lower risk of immaturity. Yet, DOR patients (here defined as women younger than 35 with a basal AMH lower than 1.2 ng/ml) represent only 5% of our dataset; therefore, further studies focused on this category of patients are suggested to confirm and expand our findings. To date, the only study reporting r-IVM results in DOR patients showed that these women collect fewer oocytes, but are also subject to lower oocyte immaturity, and a better rescue rate following r-IVM compared to controls [23].

Based on our data, time management during OS is critical, as OS protocols shorter than 11 days involved a significantly higher risk of retrieving immature oocytes. The use of a GnRH-agonist, instead, was associated with a lower risk than urinary-hCG, a topic that has been poorly investigated in the literature. This finding represents a further argument in favour of agonist protocols that adds up to existing evidence of improved OPU outcomes [24–27]. Specifically, GnRH-agonist trigger has a shorter half-life and is linked to a significantly lower risk of ovarian hyperstimulation syndrome (OHSS). Moreover, unlike hCG, GnRH-agonists induce a flare-up effect on the pituitary, leading to a more physiological release of gonadotropins (FSH and LH) that closely mimics the natural mid-cycle surge. A systematic review and meta-analysis of 29 randomized controlled trials (RCTs) demonstrated that the use of a GnRH agonist trigger, either alone or in combination with hCG, is associated with a statistically significant increase in the number of retrieved and MII oocytes compared to hCG alone [28]. The meta-analysis reported an average increase of one additional retrieved oocyte when triggering with a GnRH agonist compared to hCG. Some authors suggest that this effect may be attributed to the additional FSH surge induced by the GnRH agonist, which could play a key role in (i) promoting the resumption of oocyte meiosis, facilitating nuclear and cytoplasmic maturation; (ii) enhancing cumulus cell expansion, which is essential for optimal oocyte competence; and (iii) stimulating the release of proteolytic enzymes involved in the ovulation process, aiding in follicular rupture and oocyte release.

In ART, precise timing and stimulation protocols are essential to preserving gamete quality and reproductive competence. Our study highlights that the interval between ovulation trigger and oocyte denudation (ranging 36–44 h) has a measurable impact on oocyte maturation rates, with lower risk around 39 h and no further decrease up to 44. This total interval, however, derives from two distinct components: the time from ovulation trigger to OPU (34–38 h) and the time from OPU to denudation (2–6 h). Clearly, staying around 39 h does not imply that an early OPU can be offset by a delayed denudation, or vice versa; each interval must fall within its own physiological range. Notably, only MI rates, but not GV rates, were associated with timing variables, an expected outcome as GV oocytes cannot progress to the MII stage in vitro unless overnight culture is performed. At last, while extending the ovulation trigger to oocytes’ denudation interval beyond 39 h may decrease the risk of oocyte immaturity, it can also lead to in vitro aging of MII oocytes, potentially affecting fertilization and blastulation rates, as recently shown by our group [29]. In mammals, in fact, oocytes must be fertilized within an optimal time frame, beyond which they may undergo postovulatory aging (POA), a phenomenon that can compromise their developmental potential [30]. Combining the evidence from the present study and our previous investigation [29], denuding oocytes around 39 h after ovulation trigger (as a result of an average 35.5-h interval from ovulation trigger to OPU and of an average 3.5-h interval from OPU to oocytes’ denudation) might represent the ideal timing to maximize IVF outcomes.

Regarding the COCs retrieved at OPU, we observed that when their number is lower, equal to, or slightly higher than the number of follicles > 14 mm at the time of ovulation trigger (ratio < 1.13), the immaturity rate remains within the expected range, if not being even lower. However, when the number of retrieved COCs significantly exceeds the number of follicles > 14 mm, the immaturity rate is significantly higher, suggesting that smaller follicles are more likely to contain immature oocytes. Despite this, the risk of failing to recruit some MII oocytes must also be considered. Future research should thus focus on evaluating the developmental competence of MII oocytes in relation to follicular size at the time of retrieval, as follicle size plays a crucial role in synchronizing follicular growth during OS.

In this study, we identified a statistically robust warning limit for oocyte immaturity rate at 51%. This threshold could serve as a discriminatory criterion to identify patients who may benefit the most from rescue-IVM. As such, it lays the groundwork for further research across different patient populations to evaluate rescue-IVM as a targeted intervention in cases where oocyte maturation is significantly impaired. It should be noted that, according to the Vienna Consensus, a maturation rate below 75% is indicative of a suboptimal response to ovarian stimulation [31]; however, this threshold is not sufficient to suggest an intrinsic defect in oocyte maturation. According to Wei et al. [32], candidates suitable for rescue-IVM are patients who yield fewer than nine MII oocytes in a conventional IVF/ICSI cycle. In these patients, rescue-IVM significantly improved clinical pregnancy rates and cumulative live birth rates compared to those undergoing ICSI with MII oocytes alone (≥ 10% absolute increase). However, these improvements were not observed in patients with MII counts ≥ 9, suggesting that rescue-IVM may be particularly beneficial for cases with compromised oocyte maturation.

Studies like ours are timely, as the recent update of the Istanbul Consensus suggested that rescue-IVM could be offered to poor-prognosis patients [14]. However, a precise definition of the ideal patient profile remains lacking despite being crucial for optimizing clinical outcomes. A targeted approach to rescue-IVM not only reduces treatment costs but also streamlines laboratory workflows, facilitating more efficient resource allocation. By identifying patients who would benefit the most from this technique, clinics can enhance both cost-effectiveness and overall success rates in ART.

### Design decisions, methodological choices, and limitations

Rescue-IVM has not yet been widely adopted in clinical IVF practice worldwide, and conclusive data on its efficacy, particularly in terms of maturation, fertilization, blastulation, euploidy, and implantation rates, remains limited. However, studies have shown that oocytes matured through rescue-IVM can still reach the blastocyst stage, albeit at a lower rate, and can result in live births [33]. Based on these observations, rescue-IVM could be considered for patients with a poor prognosis, as each additional MII oocyte may improve treatment efficacy. Notably, patients retrieving fewer than five COCs and exhibiting high immaturity rates might also significantly benefit from rescue-IVM. Although this scenario is also clinically relevant, we chose not to assess it here; therefore, it warrants further investigation from future studies. Looking ahead, once robust and evidence-based guidelines are established, a careful case-by-case evaluation will remain essential to ensure optimal patient selection and clinical outcomes.

Another potential application of the oocyte immaturity rate as a KPI for future scientific and clinical advancements is the identification of patients who may benefit from in-depth genetic investigations, such as whole-exome sequencing. This approach could help uncover genetic causes of recurrent oocyte maturation defects or developmental incompetence, independent of maternal age [7, 34]. For this purpose, exceeding the more restrictive control limit (weighted average + 3 SD; in this study 67%) may be more suitable, as this use case focuses on enhancing the precision of infertility diagnosis and paving the way for targeted interventions and innovative therapeutic strategies in reproductive medicine. Conversely, for future clinical investigation of rescue-IVM, we considered the warning limit (weighted average + 2 SD) to be a more plausible threshold, as it may offer a more balanced approach between cost-effectiveness and clinical utility.

## Conclusions and future perspectives

In this study, the weighted average oocyte immaturity rate was 19%, which is consistent with findings reported in the literature [4, 5]. Our analysis outlined key factors that significantly impact oocyte maturation: (i) OS duration, as protocols shorter than 11 days were associated with higher immaturity rates; (ii) trigger type, supporting GnRH-agonist as being both safe and effective in optimizing oocyte maturation; (iii) ovulation trigger to oocytes’ denudation interval, considering that longer timings than 39 h associate with lower oocyte immaturity rates (yet, being at the risk of lower developmental competence [29]; (iv) ratio of COCs retrieved at OPU versus follicles > 14 mm at ovulation trigger > 1.13 as, while maximizing oocyte yield is essential, it must be acknowledged that smaller follicles are more likely to contain immature oocytes.

An oocyte immaturity rate exceeding 51% in patients retrieving at least five oocytes serves as a warning threshold and may represent a strong inclusion criterion for future clinical studies on rescue-IVM. This condition was observed in approximately 4% of all OPUs, with 1–2% of patients experiencing recurrent immaturity rates across multiple attempts. In addition, the fact that the rate of the affected population decreased across three attempts suggests that the low maturation rate can and should be tackled.

Significant questions remain regarding the standardization of rescue-IVM, such as (i) the choice of culture medium, (ii) the optimal length of culture, and particularly whether extended overnight culture should be considered or not, (iii) the use of time-lapse imaging versus static observations, and (iv) the sperm preparation protocol, and particularly whether the same ejaculate should be used for ICSI or a new fresh sample should be rather collected. Moreover, further research is needed to explore strategies for enhancing oocyte maturation rates, such as the potential benefits of adding culture media supplements. Identifying which patient groups benefit the most from rescue-IVM is essential, as it is to assess the cost-effectiveness of this approach. Finally, future studies should evaluate the competence of oocytes matured via rescue-IVM in terms of embryological, clinical, gestational, and postnatal outcomes to establish the safety and efficacy of this procedure in reproductive medicine.

## Supplementary information

Below is the link to the electronic supplementary material.

ESM1 (DOCX 19.8 KB)

ESM2 Definition of Weighted-Average oocyte immaturity Rate, germinal vesicle (GV) and metaphase I (MI) oocytes’ rates. The analysis identified a weighted average immaturity rate of 19%, indicating that one immature oocyte might be obtained from ≥ 5 COCs. The same value was 12% for the GV oocytes’ rate and 7% for the MI oocytes’ rate (PDF 141 KB)

ESM3 Oocyte immaturity rate after ovarian stimulation was not associated with: ranges of maternal age (A), maternal karyotype (B), main cause of female infertility (C). Kruskal Wallis tests showed no significant association (PDF 133 KB)

ESM4 Association between the oocyte immaturity rate and ovulation trigger to oocyte pick up (OPU) interval (A), and OPU to oocyte’ denudation interval (B).
*Binomial regression with logit link function was used to model the immaturity rate formulated as number of events (immature oocytes) per cohort (total collected oocytes) versus the characteristics under investigation. Non-linear associations were modelled with restricted cubic splines and model fits were compared using Akaike Information Criterion. Predicted marginal means from multivariable models were plotted to depict the associations.*
*RR, Risk Ratio *(PDF 61.3 KB)

ESM5 Variables associated with germinal vesicle (GV) oocytes’ rate. The variables significantly associated with immaturity rates include ovarian stimulation length (A), the use of a GnRH agonist trigger versus urinary hCG (u-hCG) (B), the ovulation trigger to oocytes’ denudation interval (C), and the ratio cumulus oocyte complex (COC) to follicle > 14 mm at ovulation trigger (D).
*Binomial regression with logit link function was used to model the immaturity rate formulated as number of events (immature oocytes) per cohort (total collected oocytes) versus the characteristics under investigation. Non-linear associations were modelled with restricted cubic splines and model fits were compared using Akaike Information Criterion. Predicted marginal means from multivariable models were plotted to depict the associations *(PDF 162 KB)

ESM6 Variables associated with metaphase I (MI) oocytes’ rate. The variables significantly associated with immaturity rates include ovarian stimulation length (A), the use of a GnRH agonist trigger versus urinary hCG (u-hCG) (B), the ovulation trigger to oocytes’ denudation interval (C), and the ratio cumulus oocyte complex (COC) to follicle > 14 mm at ovulation trigger (D).
*Binomial regression with logit link function was used to model the immaturity rate formulated as number of events (immature oocytes) per cohort (total collected oocytes) versus the characteristics under investigation. Non-linear associations were modelled with restricted cubic splines and model fits were compared using Akaike Information Criterion. Predicted marginal means from multivariable models were plotted to depict the associations *(PDF 162 KB)

## Data Availability

The data underlying this article are available in the article and in its online supplementary material.
