# Supplementary material for: Rates and risk factors of oocyte immaturity: toward personalized selection for rescue in vitro maturation
Source: J Assist Reprod Genet. 2025 Nov 14;43(1):167–77. doi: 10.1007/s10815-025-03722-z (PMC12831772; doi:10.1007/s10815-025-03722-z)
Supplement: Supplementary file 1 — (DOCX 19.8 KB) [file 10815_2025_3722_MOESM1_ESM.docx]

**Supplementary Table 1.**

Binomial regression with logit link function to model the immaturity rate formulated as number of events (immature oocytes) per cohort (total collected oocytes) versus the duration of ovarian stimulation in days. Non-linear associations were modelled with restricted cubic splines and model fits were compared using Akaike Information Criterion.

| *Predictor* | *Risk Ratio* | *95%CI* | *p-value* |
| --- | --- | --- | --- |
| (Intercept) | 0.29 | 0.27 – 0.31 | **<0.001** |
| Duration of ovarian stimulation [<11 days] | 0.44 | 0.39 – 0.51 | **<0.001** |
| Duration of ovarian stimulation [>11 days] | 0.92 | 0.78 – 1.09 | 0.344 |

**Supplementary Table 2.**

Binomial regression with logit link function to model the immaturity rate formulated as number of events (immature oocytes) per cohort (total collected oocytes) versus the trigger type. Non-linear associations were modelled with restricted cubic splines and model fits were compared using Akaike Information Criterion.

| *Predictor* | *Risk Ratio* | *95%CI* | *p-value* |
| --- | --- | --- | --- |
| (Intercept) | 0.21 | 0.20 – 0.21 | **<0.001** |
| Trigger type [GnRH agonist versus urinary hCG] | 0.91 | 0.88 – 0.93 | **<0.001** |

**Supplementary Table 3.**

Binomial regression with logit link function to model the immaturity rate formulated as number of events (immature oocytes) per cohort (total collected oocytes) versus the ovulation trigger to oocytes’ denudation interval in hours. Non-linear associations were modelled with restricted cubic splines and model fits were compared using Akaike Information Criterion.

| *Predictors* | *Risk Ratio* | *95%CI* | *p-value* |
| --- | --- | --- | --- |
| (Intercept) | 0.22 | 0.20 – 0.23 | **<0.001** |
| Ovulation trigger to oocytes’ denudation interval [<39 hours] | 0.77 | 0.67 – 0.88 | **<0.001** |
| Ovulation trigger to oocytes’ denudation interval [>39 hours] | 1.00 | 0.87 – 1.15 | 0.987 |

**Supplementary Table 4.**

Binomial regression with logit link function to model the immaturity rate formulated as number of events (immature oocytes) per cohort (total collected oocytes) versus the ratio cumulus oocyte complex (COC) to follicle >14 mm at ovulation trigger. Non-linear associations were modelled with restricted cubic splines and model fits were compared using Akaike Information Criterion.

| *Predictors* | *Risk Ratio* | *95%CI* | *p-value* |
| --- | --- | --- | --- |
| (Intercept) | 0.14 | 0.13 – 0.14 | **<0.001** |
| Ratio COC to fol1icle >14 mm at ovulation trigger [<1.13] | 2.90 | 2.54 – 3.32 | **<0.001** |
| Ratio COC to fol1icle >14 mm at ovulation trigger [>1.13] | 1.68 | 1.52 – 1.84 | **<0.001** |
