# Supplementary figures and images for: Rates and risk factors of oocyte immaturity: toward personalized selection for rescue in vitro maturation
Source: J Assist Reprod Genet. 2025 Nov 14;43(1):167–77. doi: 10.1007/s10815-025-03722-z (PMC12831772; doi:10.1007/s10815-025-03722-z)

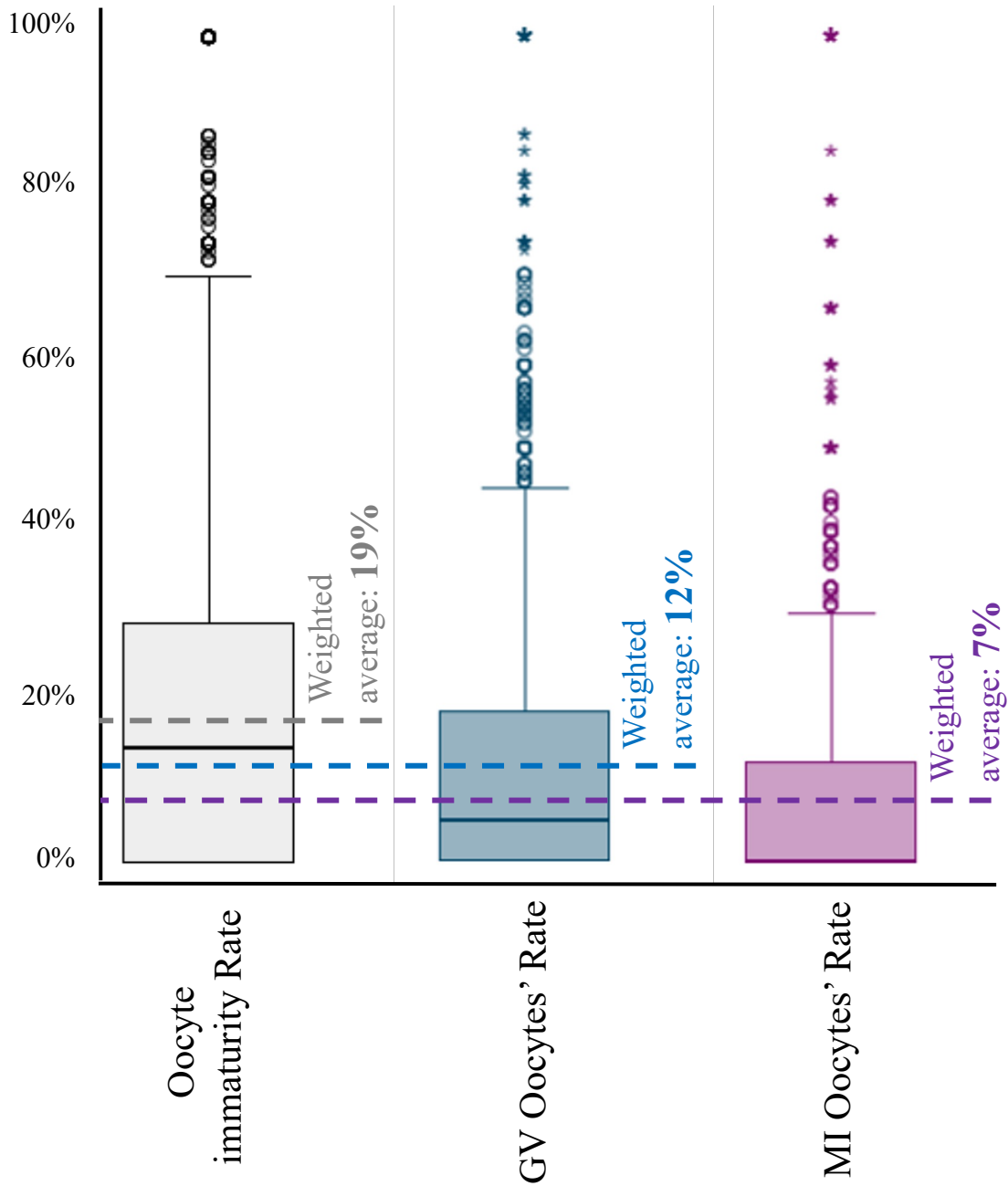

Supplement: Supplementary file 2 — Definition of Weighted-Average oocyte immaturity Rate, germinal vesicle (GV) and metaphase I (MI) oocytes’ rates. The analysis identified a weighted average immaturity rate of 19%, indicating that one immature oocyte might be obtained from ≥ 5 COCs. The same value was 12% for the GV oocytes’ rate and 7% for the MI oocytes’ rate (PDF 141 KB) [file 10815_2025_3722_MOESM2_ESM.pdf]

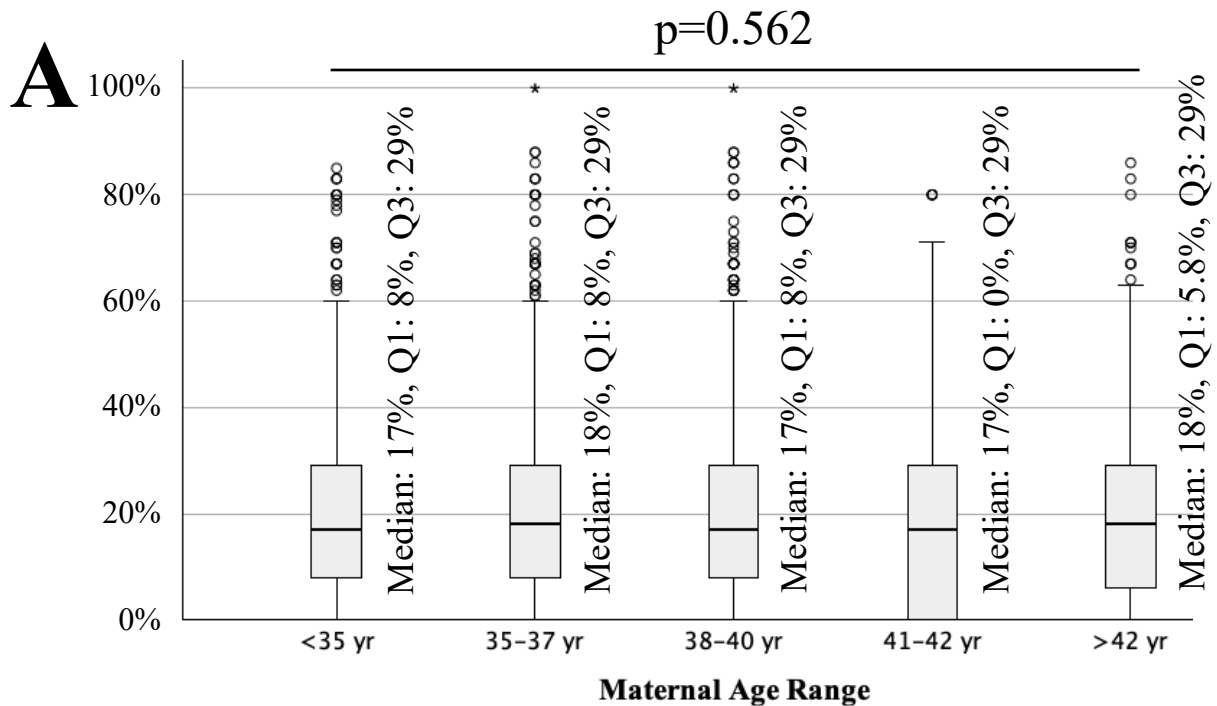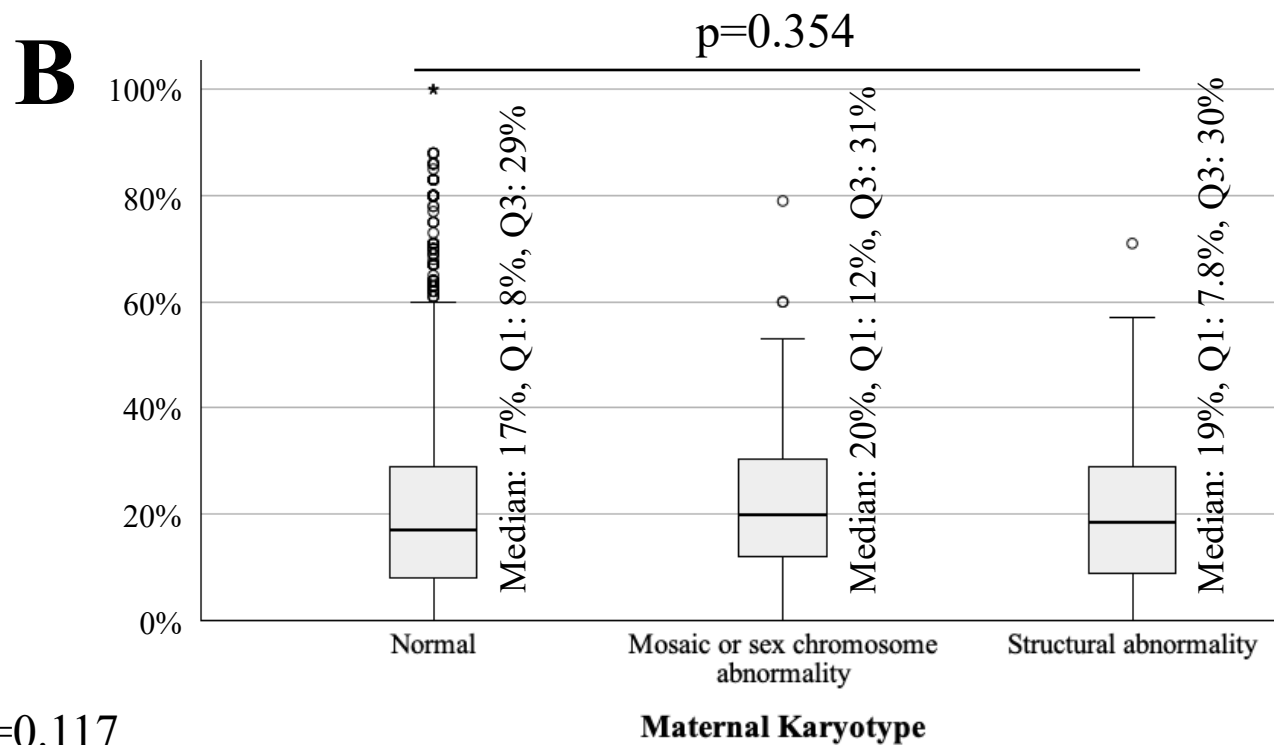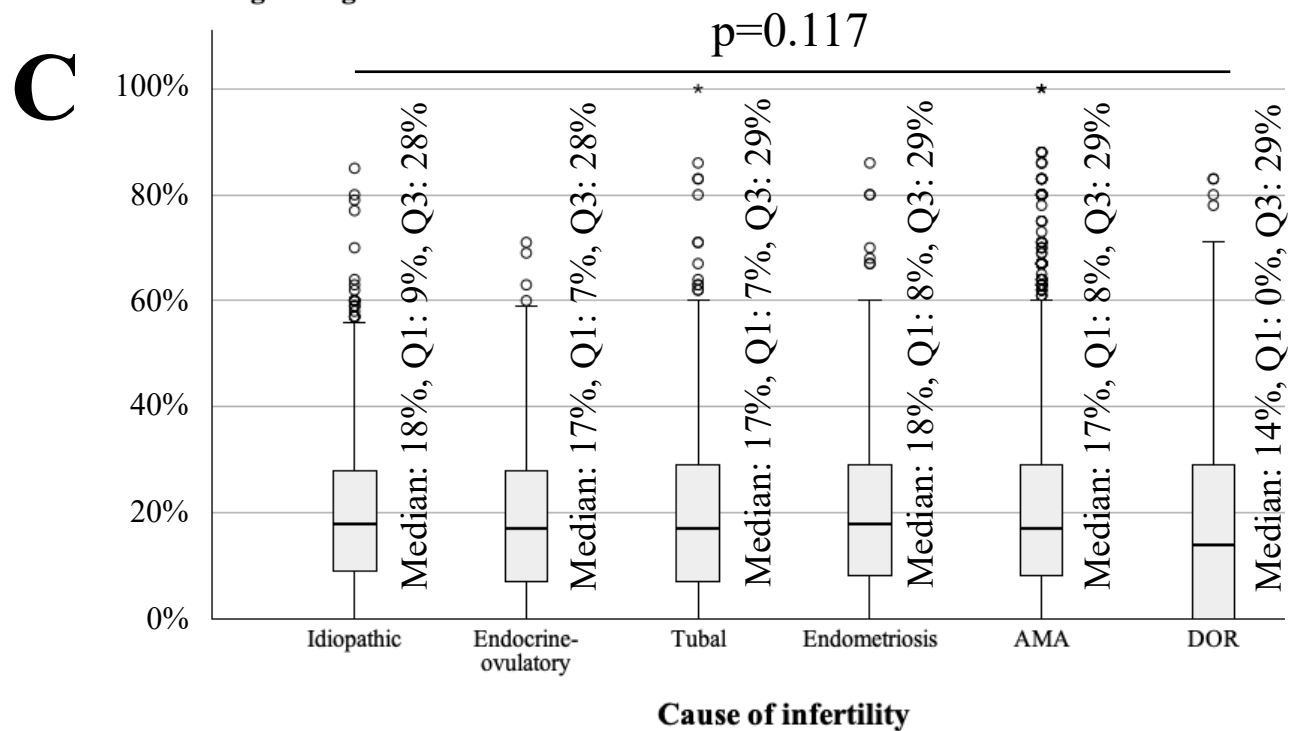

Supplement: Supplementary file 3 — Oocyte immaturity rate after ovarian stimulation was not associated with: ranges of maternal age (A), maternal karyotype (B), main cause of female infertility (C). Kruskal Wallis tests showed no significant association (PDF 133 KB) [file 10815_2025_3722_MOESM3_ESM.pdf]

**A**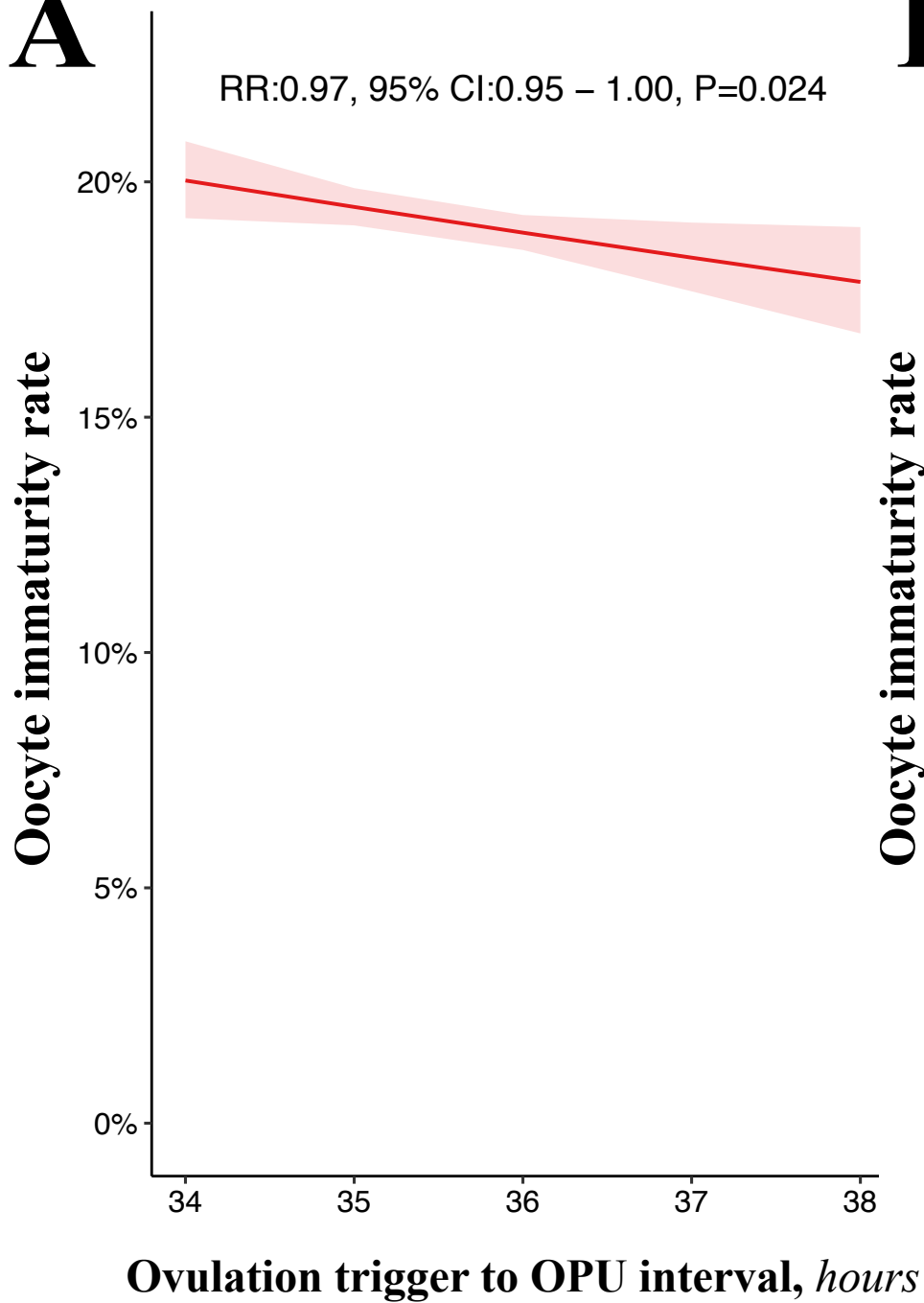**B**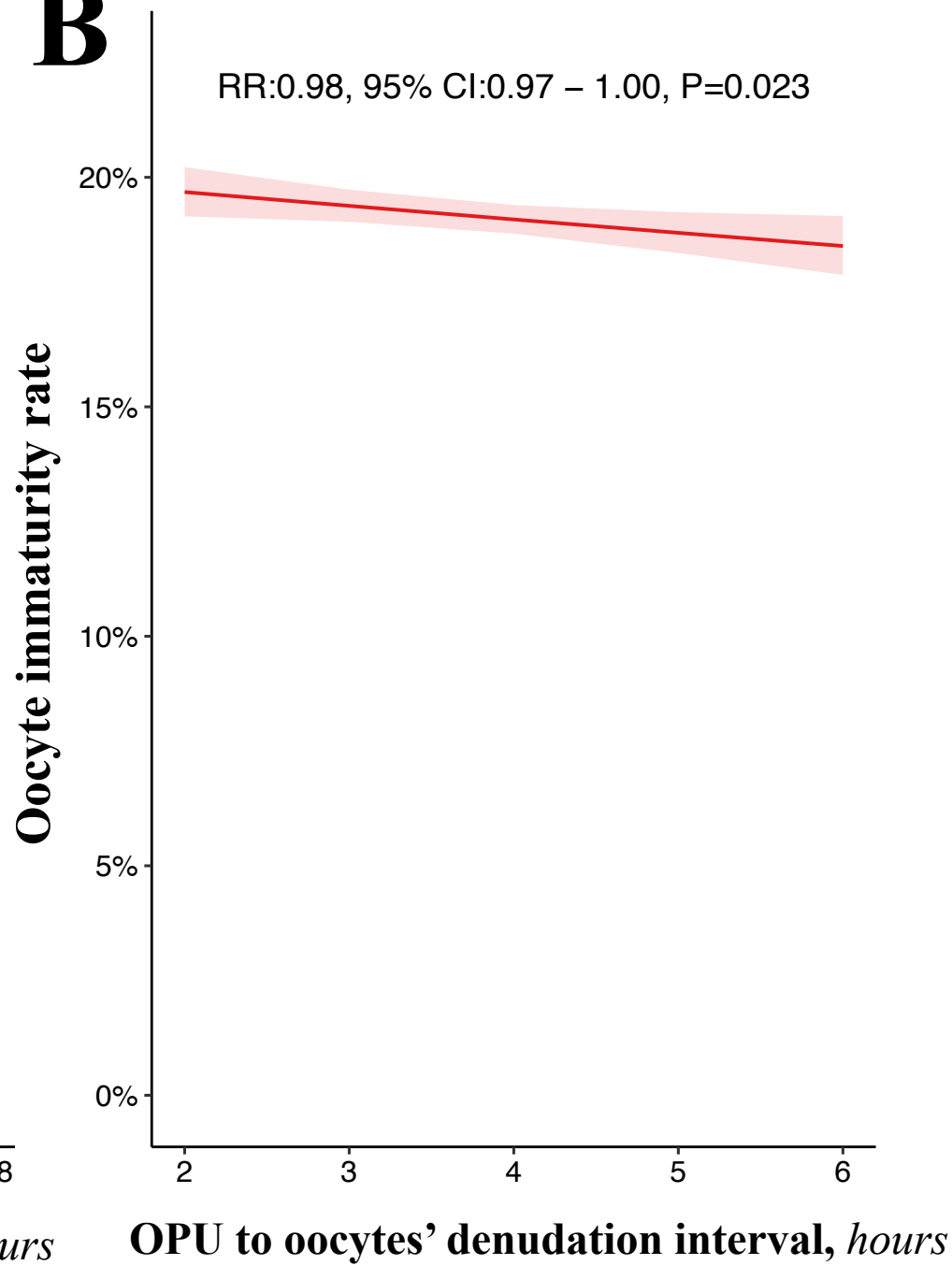

Supplement: Supplementary file 4 — Association between the oocyte immaturity rate and ovulation trigger to oocyte pick up (OPU) interval (A), and OPU to oocyte’ denudation interval (B). Binomial regression with logit link function was used to model the immaturity rate formulated as number of events (immature oocytes) per cohort (total collected oocytes) versus the characteristics under investigation. Non-linear associations were modelled with restricted cubic splines and model fits were compared using Akaike Information Criterion. Predicted marginal means from multivariable models were plotted to depict the associations. RR, Risk Ratio (PDF 61.3 KB) [file 10815_2025_3722_MOESM4_ESM.pdf]

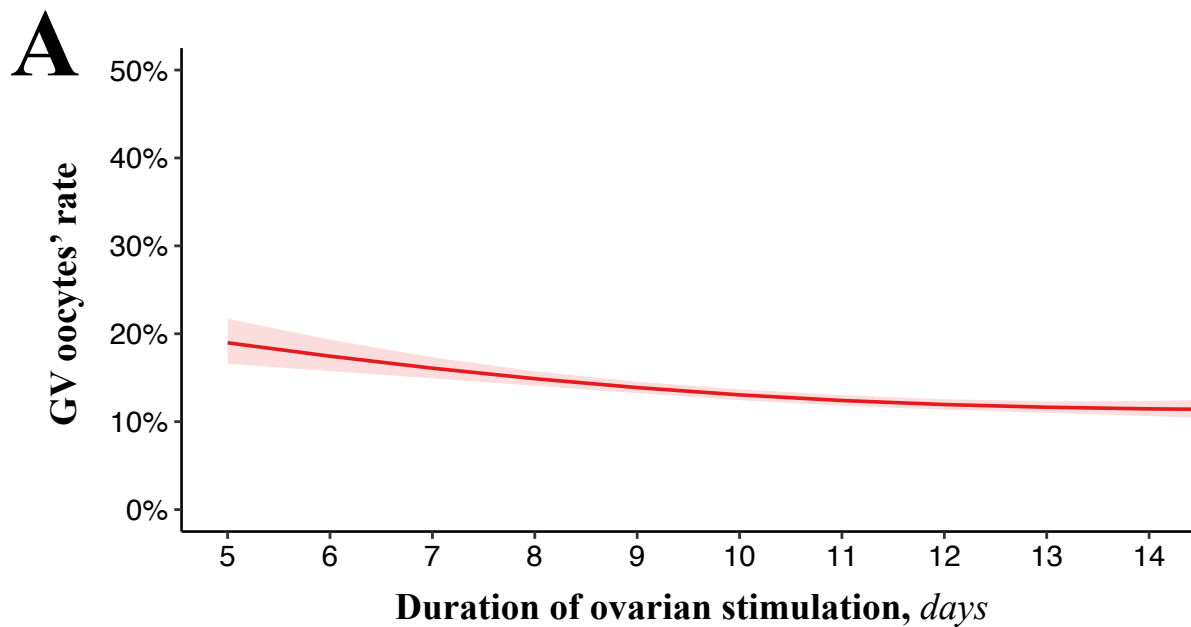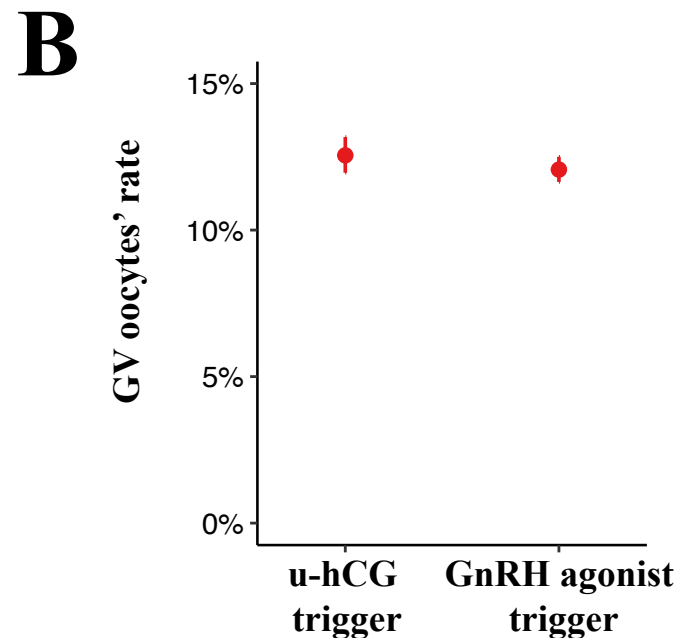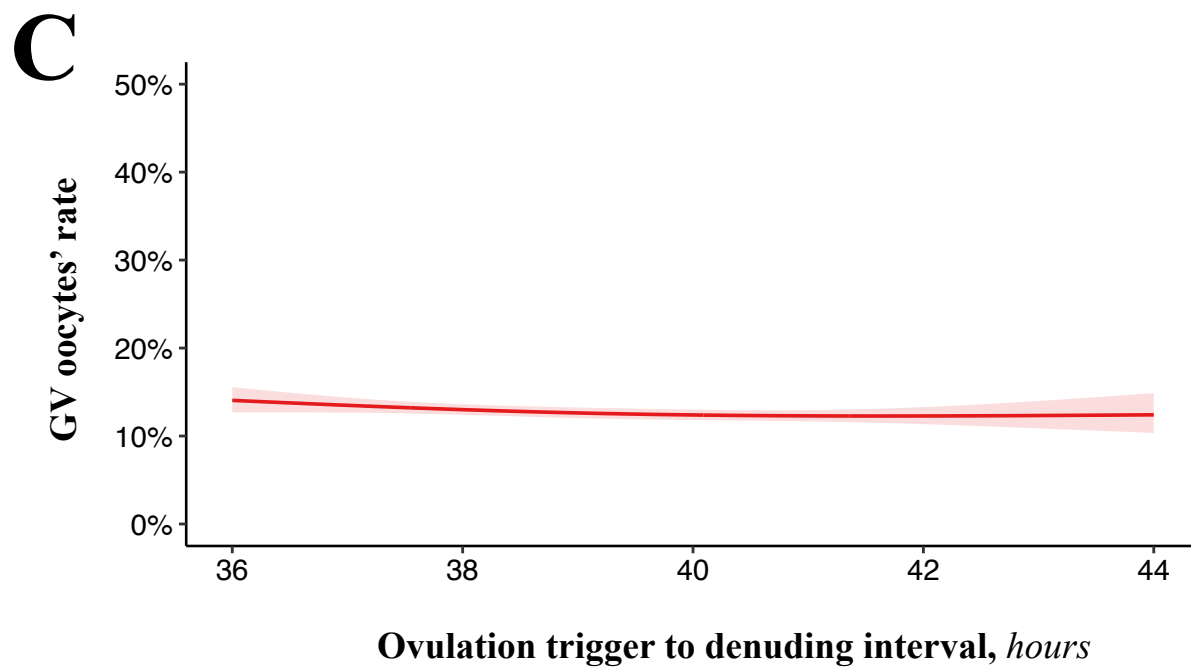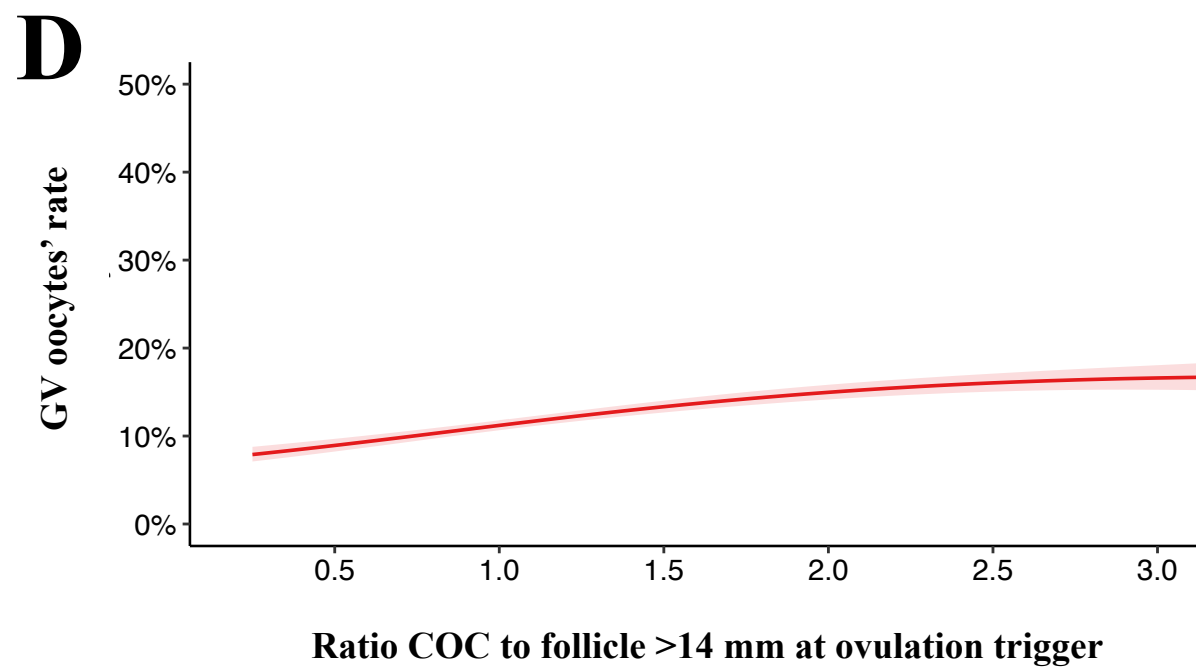

Supplement: Supplementary file 5 — Variables associated with germinal vesicle (GV) oocytes’ rate. The variables significantly associated with immaturity rates include ovarian stimulation length (A), the use of a GnRH agonist trigger versus urinary hCG (u-hCG) (B), the ovulation trigger to oocytes’ denudation interval (C), and the ratio cumulus oocyte complex (COC) to follicle > 14 mm at ovulation trigger (D). Binomial regression with logit link function was used to model the immaturity rate formulated as number of events (immature oocytes) per cohort (total collected oocytes) versus the characteristics under investigation. Non-linear associations were modelled with restricted cubic splines and model fits were compared using Akaike Information Criterion. Predicted marginal means from multivariable models were plotted to depict the associations (PDF 162 KB) [file 10815_2025_3722_MOESM5_ESM.pdf]

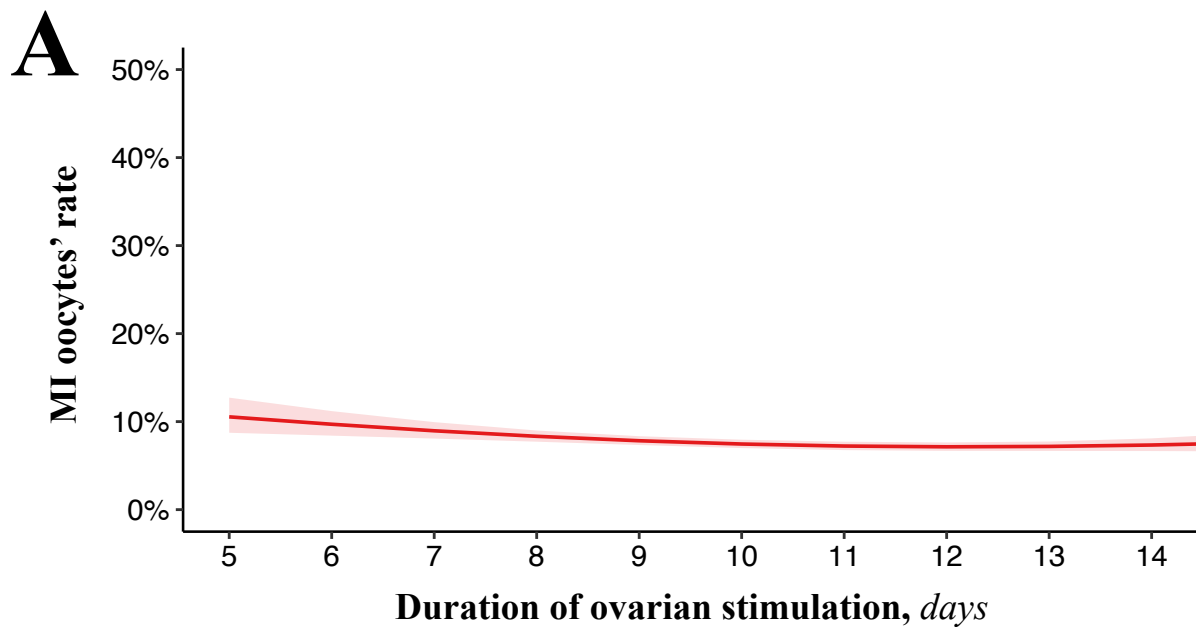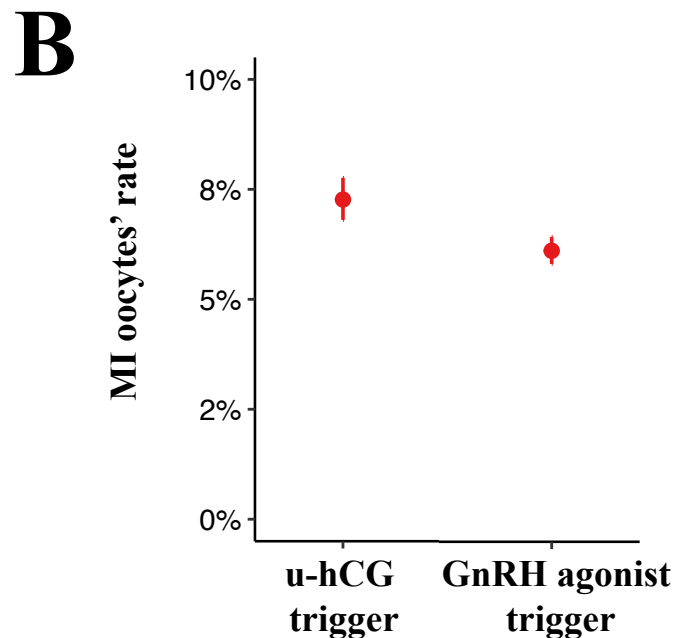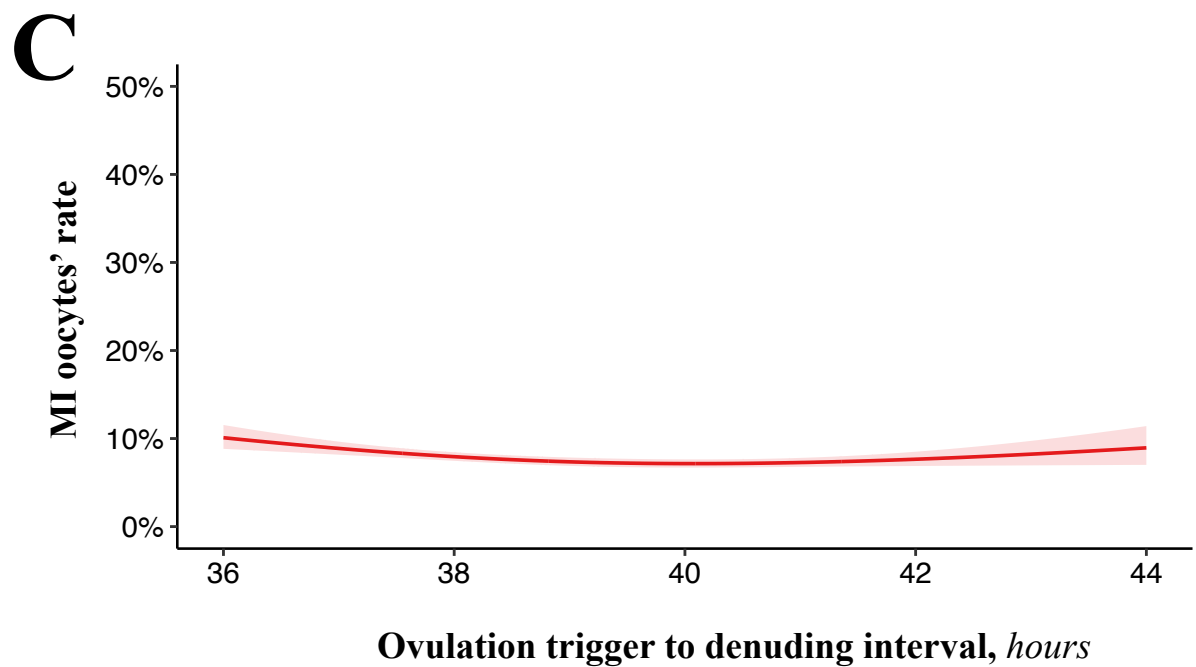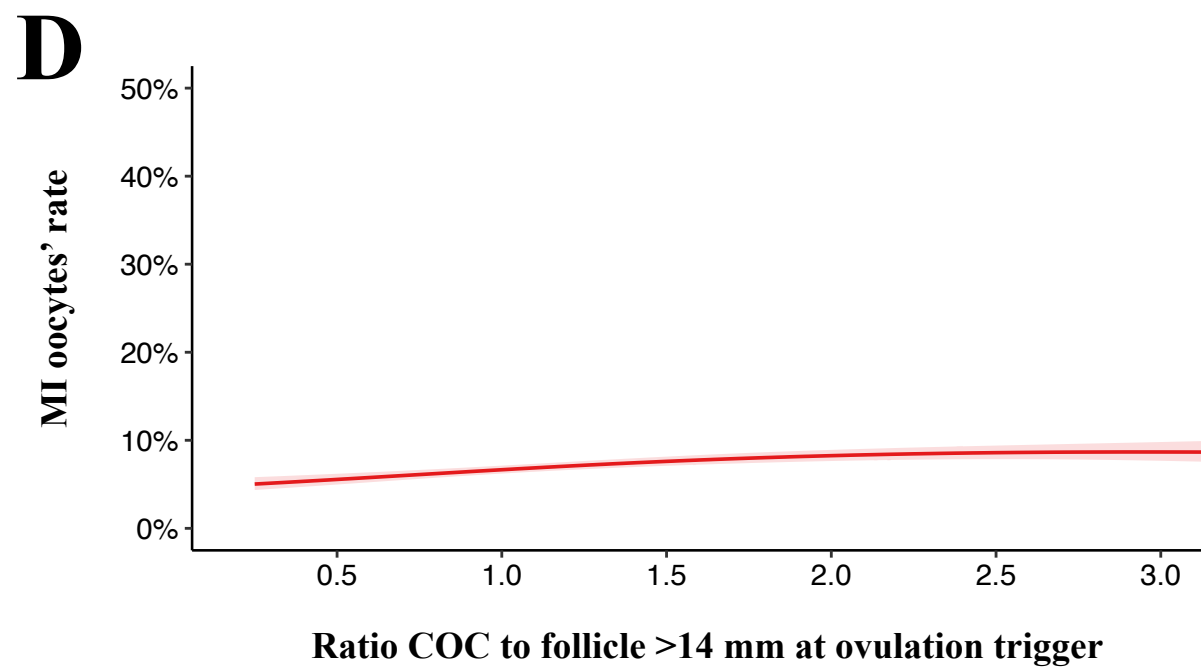

Supplement: Supplementary file 6 — Variables associated with metaphase I (MI) oocytes’ rate. The variables significantly associated with immaturity rates include ovarian stimulation length (A), the use of a GnRH agonist trigger versus urinary hCG (u-hCG) (B), the ovulation trigger to oocytes’ denudation interval (C), and the ratio cumulus oocyte complex (COC) to follicle > 14 mm at ovulation trigger (D). Binomial regression with logit link function was used to model the immaturity rate formulated as number of events (immature oocytes) per cohort (total collected oocytes) versus the characteristics under investigation. Non-linear associations were modelled with restricted cubic splines and model fits were compared using Akaike Information Criterion. Predicted marginal means from multivariable models were plotted to depict the associations (PDF 162 KB) [file 10815_2025_3722_MOESM6_ESM.pdf]
